# *Brachypodium distachyon* exhibits compatible interactions with *Oculimacula* spp. and *Ramularia collo-cygni*, providing the first pathosystem model to study eyespot and ramularia leaf spot diseases

**DOI:** 10.1111/ppa.12114

**Published:** 2013-08-06

**Authors:** A Peraldi, L L Griffe, C Burt, G R D McGrann, P Nicholson

**Affiliations:** Department of Crop Genetics, John Innes CentreColney Lane, Norwich, NR4 7UH, UK

**Keywords:** *Oculimacula acuformis*, *Oculimacula yallundae*, plant–pathogen interaction

## Abstract

*Brachypodium distachyon* (Bd) has established itself as an essential tool for comparative genomic studies in cereals and increasing attention is being paid to its potential as a model pathosystem. Eyespot and ramularia leaf spot (RLS) are important diseases of wheat, barley and other small-grain cereals for which very little is known about the mechanisms of host resistance despite urgent requirements for plant breeders to develop resistant varieties. This work aimed to test the compatibility of interaction of two Bd accessions with the cereal pathogens *Oculimacula* spp. and *Ramularia collo*-*cygni*, the causal agents of eyespot and RLS diseases, respectively. Results showed that both Bd accessions developed symptoms similar to those on the natural host for all pathogen species tested. Microscopy images demonstrated that *R. collo-cygni* produced secondary conidia and both *Oculimacula* spp. formed characteristic infection structures on successive tissue layers. Visual disease assessment revealed that quantitative differences in disease severity exist between the two Bd accessions. The results presented here provide the first evidence that Bd is compatible with the main causal agents of eyespot and RLS diseases, and suggest that future functional genetic studies can be undertaken to investigate the mechanisms of eyespot and RLS disease resistance using Bd.

## Introduction

*Brachypodium distachyon* (Bd) is an annual monocotyledonous plant of the grass family that was proposed over a decade ago as a new model for functional genomics in temperate grasses and cereals (Draper *et al*., 2001). Since Bd was first suggested as a model plant, numerous publications have reviewed the physiological and genetic advantages of Bd as a comparative and functional genetic model and its potential relevance for research on small-grain cereals (Brkljacic *et al*., 2011; Mur *et al*., 2011). The undemanding growth requirements of Bd, its short stature (about 30 cm at maturity), rapid life cycle (8–10 weeks), self-fertility, a small fully sequenced diploid genome (about 272 Mbp for the Bd21 diploid accession) and high degree of chromosomal synteny with wheat and barley (IBI, 2010) represents all the desirable physiological and genetic features of a powerful plant model. The higher degree of chromosomal synteny shared between wheat and Bd compared to other small-grain cereals allowed the fine-mapping of the complex *Ph1* locus region in wheat (Griffiths *et al*., 2006), demonstrating the potential of Bd as a genetic tool.

Publication of the entire genome sequence of the inbred line Bd21 in 2010 (IBI, 2010) has enabled comparative genomic studies of entire signalling pathways (Higgins *et al*., 2012) and genome-wide analysis of Bd gene families such as nucleotide-binding site disease resistance genes (Tan & Wu, 2012), lipoxygenases (Feng *et al*., 2012) and WRKY transcription factors (Tripathi *et al*., 2012). Recent efforts have been devoted to adapting methods and protocols routinely used in research on cereals to the Bd system and expand the means by which to conduct research transferable from the model to cereals.

Bd has potential as a pathosystem model for diseases of small-grain cereals. Hence, a number of economically significant fungal diseases such as those caused by the rice blast fungus (*Magnaporthe grisea*; Routledge *et al*., 2004), yellow rusts (*Puccinia striiformis* f. sp. *hordei* and f. sp. *triticae*; Draper *et al*., 2001 and *Puccinia brachypodii*; Barbieri *et al*., 2011), and *Fusarium* species (*F. graminearum* and *F. culmorum*; Peraldi *et al*., 2011) have been reported to establish a compatible interaction with Bd that mirrors infection of the respective cereal host. Recently, a biparental population developed from a cross between the Bd accessions Bd21 and Bd3-1 was used to fine-map a resistance gene effective against *Barley stripe mosaic virus* (BSMV; Cui *et al*., 2012), whilst a compatible interaction between Bd and arbuscular mycorhizzal fungi has also been reported (Hong *et al*., 2012). Therefore, Bd is a useful model for studying the biology of multiple plant–microbe interactions.

Eyespot is a stem base disease of wheat and other small-grain cereals caused by two closely related fungal species: *Oculimacula yallundae* (formerly *Tapesia yallundae*) and *Oculimacula acuformis* (formerly *Tapesia acuformis;* Crous *et al*., 2003). Eyespot is more prevalent in cool and wet regions of the world and can lead to significant yield reduction due to reduced nutrient transport at the stem base and predisposition to lodging (Lucas *et al*., 2000). Visual symptoms are characterized by elliptical-shaped lesions with dark centres forming on the leaf sheaths and culms near ground level. One of the most distinctive microscopic infection structures produced by the eyespot-causing fungi is the formation of multicellular aggregates on the host leaf sheath, termed infection plaques. Branching and aggregation of hyphae provide the source of subsequent infection hyphae which penetrate the host cuticle and epidermal cell wall (Daniels *et al*., 1991). Only three sources of resistance have been identified in commercial wheat varieties: *Pch1* (Worland *et al*., 1988), *Pch2* (de la Peña *et al*., 1996) and a QTL on chromosome 5A (Burt *et al*., 2010a). However, these resistance loci have drawbacks such as linkage with deleterious traits (Koen *et al*., 2002) or limited effectiveness against the disease (Burt *et al*., 2010b).

*Ramularia collo-cygni* (formerly *Ophiocladium hordei*; Sutton & Waller, 1988) is the causal agent of ramularia leaf spot (RLS) disease of barley (Sachs *et al*., 1998). Infection by *R. collo-cygni* induces necrotic spotting of the foliage and premature senescence, leading to loss of green leaf area that can result in substantial yield losses (Walters *et al*., 2008). One of the most characteristic features of the *R. collo-cygni* life cycle is the production of curved conidiophores which resemble a swan's neck. Little is known about the mechanisms of resistance against RLS, as this emerging disease has only recently become problematic in northern Europe (Walters *et al*., 2008). However, research has indicated that the development of RLS symptoms is affected by light levels, being more severe under higher light intensities (Makepeace *et al*., 2008).

The work presented in this manuscript demonstrates the potential for Bd to be used as a reference pathosystem model for a wider range of cereal pathogens than originally proposed by demonstrating the compatibility of this model plant with the wheat pathogens *O. yallundae* and *O. acuformis* and the barley pathogen *R. collo-cygni*.

## Material and methods

### Maintenance and preparation of fungal inoculum

Inoculum of *O. yallundae* and *O. acuformis* from the JIC culture collection were prepared on V8 agar (9 g bactoagar, 50 mL V8 vegetable juice in 450 mL deionized water) in a 16°C growth cabinet for 21 days. For each species, a mixture of six different isolates was used for inoculations as detailed by Chapman *et al*. (2008).

*Ramularia collo-cygni* inoculum was prepared based on the method described by Makepeace *et al*. (2008) with the following modifications. Inoculum of isolate Rcc09B4, from Scotland, was grown in potato dextrose broth (PDB) liquid culture prepared by adding a 5 mm agar plug excised from a 2-week-old potato dextrose agar culture plate. Liquid cultures were supplemented with 10 *μ*g mL^−1^ streptomycin and incubated at 20°C for 14 days under constant agitation at 175 rpm on an orbital shaker (New Brunswick Scientific Co.) in the dark.

### *Brachypodium* lines and growth conditions

Bd lines Bd21 and Bd3-1 (Vogel *et al*., 2006) were used throughout and were germinated as detailed by Peraldi *et al*. (2011). In all experiments, each individual pot (8 × 8 × 10 cm) was filled with 50% peat and sand mixed with 50% John Innes no. 2 loam compost (William Sinclair Horticulture Ltd), containing a single Bd21 and a single Bd3-1 plant. All plants were grown in a controlled environment chamber exposed to a 16/8 h light/dark cycle, with a relative humidity (RH) of 70% with different light intensity and temperature regimes depending on the test performed. Two independent *R*. *collo*-*cygni* infection tests were performed on plants grown at 17°C in the light and 12°C in the dark. In a first test, a set of 15 plants per accession was each grown under two different light intensity regimes, low light (150–200 *μ*mol m^−2^ s^−1^) and high light (400–450 *μ*mol m^−2^ s^−1^), for 3 weeks before spray inoculation, after which all plants were returned to the low light intensity regime. In a second experiment, a total of 30 plants per accession were grown under high light regime prior to spray inoculation and then separated into two groups: one group maintained in high light and the other group moved to low light conditions, similar to those used above. For the eyespot infection tests, two independent experiments were performed with plants grown at constant temperatures; one test was carried out at 10°C and one at 16°C, to assess the impact of different temperature regimes on the plant–pathogen interaction. As no significant difference in disease scores was observed between the different temperatures (*P *=* *0·291), the data from the two experiments was combined. In all experiments, three pots per inoculum or treatment were left uninoculated to serve as control and 12 pots were dedicated for each infection test.

### Eyespot inoculation of *Brachypodium distachyon* and visual disease scoring method

Plastic collars (3 cm long and 0·5 mm internal diameter) were placed around the stem bases of 1-week-old Bd21 and Bd3-1 plants. After a further week, inoculum was prepared by homogenizing the V8 agar and associated fungal colonies with water (2:1) for *O. acuformis* and *O. yallundae* inoculum separately. Individual stem bases were inoculated by injecting 2 mL of the inoculum mix into each collar using a pipette. Plants were returned to incubate at 10 or 16°C for 6 weeks before plants were harvested and scored. The architecture of the Bd stem base differs from that of wheat and barley in that tillers only emerge from the base of the main stem at an early stage, but as the mesocotyl grows, tillering continues to occur above successive nodes along the stem. Therefore, a simple visual disease scoring system was adapted from the method used in wheat (Scott & Hollins, 1974) to account for the architecture of the Bd stem base. The incidence of the disease was calculated as the number of tillers exhibiting characteristic eyespot lesions divided by the total number of tillers. The severity of the disease was calculated as the number of layers with symptoms (leaf sheath and stem) divided by the number of infected tillers. A disease index was calculated by multiplying the incidence score by the severity score.

### Ramularia leaf spot inoculation of *Brachypodium distachyon* and visual disease scoring method

Three 14-day-old 200 mL liquid cultures were combined in a blender to form a slurry containing hyphal fragments. Prior to inoculation, a single 20 *μ*L drop of Tween 20 was added to 50 mL inoculum. Plants were sprayed evenly at a rate of 10 mL inoculum per 50 plants using an airbrush sprayer (Clarke International). Inoculated plants were kept covered under plastic lids in the dark for 48 h post-inoculation before being returned to the controlled environment cabinet at the specified light regimes. The response of Bd to *R*. *collo*-*cygni* infection was scored 21 days following spray inoculation and expressed as a percentage of the total leaf area covered with necrotic spots and as the percentage of leaf area that had developed chlorosis.

### Light microscopy of fungal development in *Brachypodium distachyon* tissue

Bd3-1 leaf sections and leaf sheaths were cleared in 70% ethanol at 70°C for 1 h to remove chlorophyll. Samples were stained for 1 min in aniline blue (0·1%) in lactoglycerol (1:1:1, lactic acid:glycerol:H_2_O) and mounted in 40% glycerol. Bd3-1 leaf sections infected with *R*. *collo*-*cygni* were viewed with a Leica DM6000 light microscope and photographed with a DFC 360 FX digital camera. Bd3-1 leaf sheath samples infected with *O. yallundae* were stored in lactoglycerol (1:1:1) for long term storage and, on the day of observation, were stained for 1 min in 0·1% aniline blue and mounted on microscope slides before observation under a Nikon Eclipse 800 microscope and were photographed with a Pixera Pro ES 600 digital camera.

### Confocal microscopy of *O. yallundae* infection of *Brachypodium distachyon* leaf sheaths

One day prior to microscope observation, sections of *O*. *yallundae* infected leaf sheath were delicately transferred into small Petri dishes (Sterilin; 30 mm diameter) containing sterile distilled water (SDW) and rinsed for 10 min on an orbital shaker three times. Samples were then incubated on the orbital shaker for 7 min in a 0·1 m Tris.HCl solution amended with 0·01% Uvitex-2B (Polyscience Inc.), a chitin-specific fluorescent dye, with the pH adjusted to 5·8 (100 mL 0·1 m Tris.HCl pH 5·8 + 0·01 mg Uvitex). The Uvitex solution was discarded and samples were rinsed three times in SDW, for 10 min each, on the orbital shaker. SDW was replaced with 25% glycerol and shaking continued for 20 min, after which samples were stored in the dark in 40% glycerol overnight. Samples were mounted on a microscope slide in 40% glycerol and observed using a Leica SP5 (II) confocal microscope, excited with a 405 nm laser diode and detected at 505–555 nm. Autofluorescence of cell walls was detected at 600–700 nm. Stacks and lateral views of confocal image series were obtained using the Fiji image analysis software (Schindelin *et al*., 2012).

### Statistical analysis

Visual disease scores were analysed by generalized linear modelling (GLM) using GenStat v. 9.1 (Lawes Agricultural Trust, Rothamsted Experimental Station, UK). Unpaired *t*-tests calculated within the GLMs were used to assess differences in disease symptom severity between Bd21 and Bd3-1 for *O. acuformis* and *O. yallundae* inoculations independently. Additionally, differences were tested between Bd21 and Bd3-1 for levels of *R. collo-cygni* disease severity and to test the effect of the two light treatments on *R. collo-cygni* symptom development.

## Results

### Interaction between *Oculimacula* species and Bd

**Figure 1:**
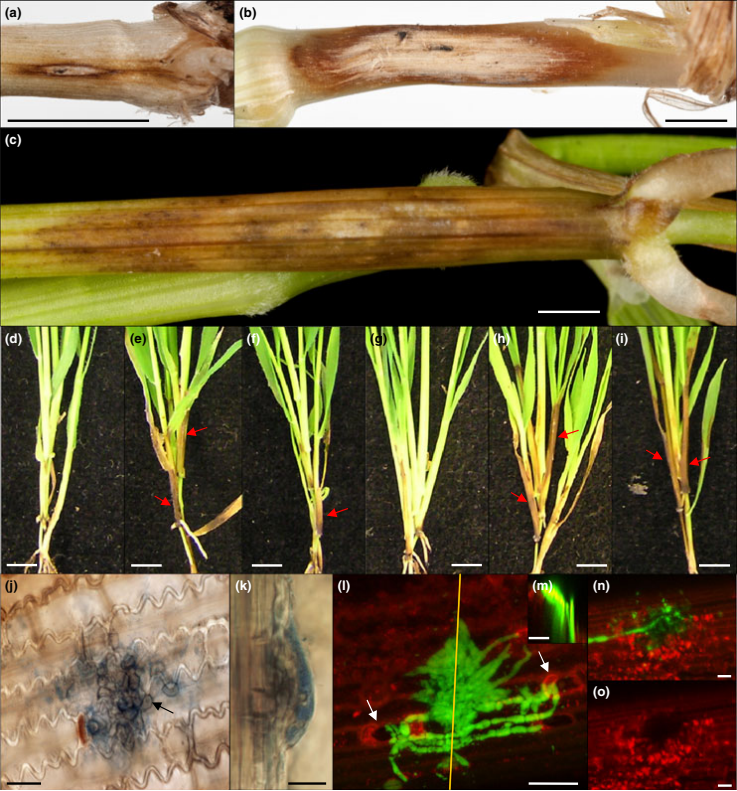
Eyespot disease symptoms expressed on *Brachypodium* Bd21 and Bd3-1 plants following stem base infection with *Oculimacula yallundae* and *Oculimacula acuformis*. (a) Mild eyespot symptom on Chinese Spring wheat stem base. (b) Severe eyespot symptom on Chinese Spring wheat stem base. Scale bars = 1 cm. (c) Eye-shaped lesion formed on Bd3-1 stem base, 28 days following infection with *O. yallundae*. Scale bar = 0·1 cm. (d–f) Disease symptom expressed on Bd21 plants, 44 days following (d) mock inoculation, (e) *O. acuformis* inoculation, (f) *O. yallundae* inoculation. (g–i) Disease symptom expressed on Bd3-1 plants, 44 days following (g) mock inoculation, (h) *O. acuformis* inoculation, (i) *O. yallundae* inoculation. (d–i) Arrows indicate eyespot symptoms. Scale bars = 1 cm. (j) Light microscope image of fungal multicellular aggregate formed on Bd3-1 leaf sheath epidermis, stained with 0·01% aniline blue, 28 days following *O. yallundae* infection. Arrow indicates pin-hole. (k) Light microscope image of eyespot infection plaque formed on Bd3-1 leaf sheath epidermis, stained with 0·01% aniline blue, 28 days following *O. yallundae* infection. (j–k) Scale bar = 10 *μ*m. (l) Composite confocal microscope image of Uvitex-stained infection plaque formed on Bd3-1 leaf sheath epidermis, 44 days following *O. yallundae* infection. Arrows indicate fungal attempt to invade stomatal cavity. (m) Lateral view of (l) showing hyphal projections underneath *O. yallundae* infection plaque. (n) Composite confocal microscope image of Uvitex-stained infection plaque connected to hyphae, formed on Bd3-1 leaf sheath epidermis, 44 days following *O. yallundae* infection. (o) Composite confocal microscope image of (n) displaying red channel to show loss of autofluorescence of epidermal cells underneath infection plaque. (l–o) Scale bars = 20 *μ*m.

**Figure 2:**
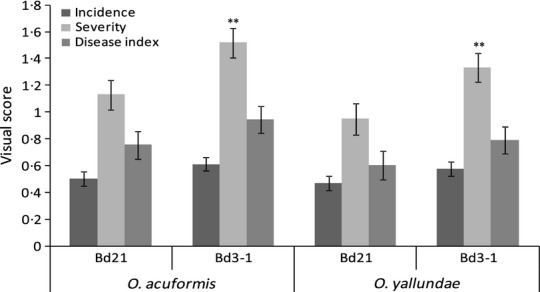
Comparison of the different visual disease scoring systems for eyespot infection on Bd21 and Bd3-1 plants, 44 days following *Oculimacula acuformis* and *Oculimacula yallundae* infection. **P-value < 0·01.

Stem base infection of Bd21 and Bd3-1 accessions with *O. yallundae* and *O*. *acuformis* resulted in development of honey-brown to amber-brown lesions on the leaf sheath immediately above the lower nodes. Eye-shaped lesions, reminiscent of those observed on wheat (Fig.1a,b), were observed on the leaf sheath of Bd3-1 plants 28 days following infection with *O. yallundae* (Fig.1c) as well as *O. acuformis* (results not shown). However, the colouration and shape of the observed lesions varied greatly depending on the combination of pathogen species and Bd accession (compare Fig.1d with 1e,f and 1g with 1h,i). Leaf sheaths of Bd21 plants developed less intensely coloured disease symptoms than those on Bd3-1 plants, regardless of the inoculated fungal species (Fig.1e,h compared to 1f,i, respectively). Disease symptoms induced by *O. acuformis* were typically darker than those induced by *O. yallundae*, regardless of Bd accession. Observation under a light microscope of Bd3-1 leaf sheath displaying eye-shaped lesions 28 days following *O. yallundae* infection revealed the presence of multicellular hyphal aggregates on the host epidermal surface (Fig.1j). Hyphal aggregate structures were observed on the epidermal surface of external as well as inner leaf sheaths, for all Bd accession and eyespot species combinations (results not shown). Localized amber-brown colouration of single cells near these aggregates was observed, although discoloration was not always associated with the presence of these fungal structures (results not shown). The hyphal aggregates appeared to be made up of lobe-shaped structures resembling appressoria (Fig.1j) and a central ‘pin-hole’ was observed at the centre of many of these structures. A transverse view through these multicellular aggregates revealed a semi-ovoid shape, tightly attached to the host epidermal cells, which appeared to be formed below the cuticle (Fig.1k). Tissue samples of Bd3-1 leaf sheath were stained with Uvitex 2-B, 44 days post-inoculation (dpi) with *O. yallundae* and observed under a confocal microscope. Multicellular aggregates were observed tightly anchored to the host epidermis from which adventitious hyphae grew outward (Fig.1l). In some instances, adventitious hyphae were observed in close contact with open stomata, apparently revealing fungal attempts to invade the stomatal cavities (Fig.1l, arrows). A lateral view of the confocal image series at this site revealed a chitin-specific signal projecting below the multicellular aggregate, suggesting that the fungus had succeeded in penetrating beneath the surface (Fig. 1m). In addition, at many sites with multicellular aggregates, epidermal cells exhibited a loss of autofluorescence directly beneath the fungal structure when separating the two fluorescent channels (Fig.1n compared to 1o). Visual disease scoring of *O. acuformis* and *O. yallundae* stem base infection of Bd21 and Bd3-1 plants was undertaken to ascertain whether the two accessions are similarly susceptible to both species of pathogen. Data from two independent experiments, run at either 10 or 16°C, were combined for analysis because no significant difference in the three types of disease score was observed between the experiments conducted at the two different temperatures (*P *=* *0·291). Bd3-1 was more susceptible to eyespot than Bd21 (Fig.2), as determined by the significantly higher severity score observed for Bd3-1 compared to Bd21 when inoculated with either *O. acuformis* (*P *=* *0·004) or with *O. yallundae* (*P *=* *0·004). However, no significant differences between Bd21 with Bd3-1 were observed for the incidence score or disease index when infected with either *O. acuformis* or *O. yallundae* (Fig.2).

### Interaction between *Ramularia collo-cygni* and Bd

**Figure 3:**
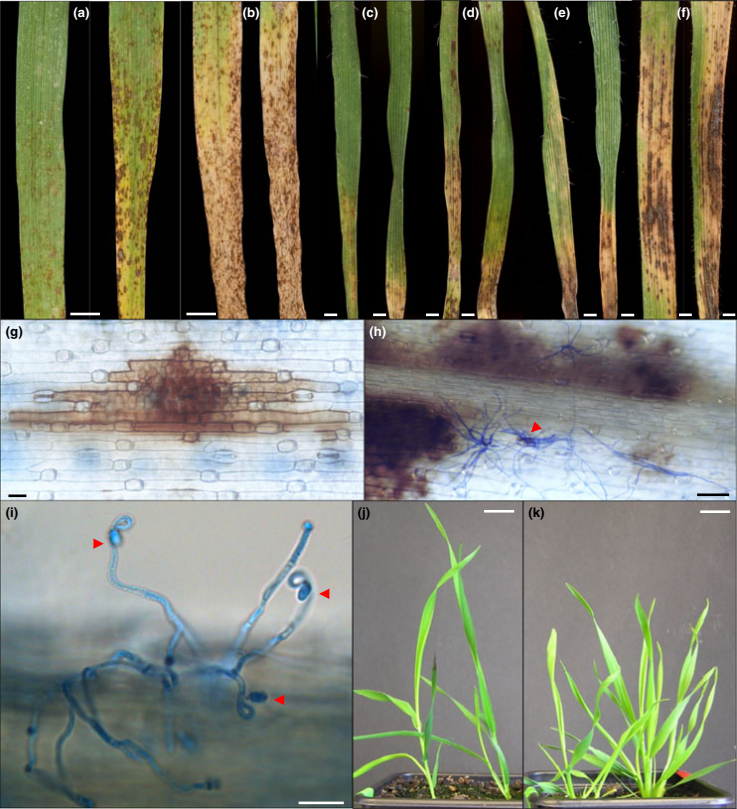
Analysis of ramularia leaf spot symptom development following spray inoculation on low and high light-treated *Brachypodium* plants. (a–b) Ramularia leaf spot symptoms on Golden Promise barley leaves, (a) 10 dpi and (b) 15 dpi. Scale bars = 0·5 cm. (c–f) Comparison of the ramularia leaf spot symptoms developed on the leaves of Bd21 (c) and Bd3-1 (d) plants exposed to low light pretreatment and leaves of Bd21 (e) and Bd3-1 (f) plants exposed to high light pretreatment, 21 days following spray inoculation with Rcc09B4. Scale bars = 0·1 cm. (g) Light microscope image of a necrotic lesion developed on the abaxial side of a leaf of Bd3-1 plant exposed to high light pretreatment, stained with 0·01% aniline blue, 21 days following spray infection with Rcc09B4. Scale bar = 20 *μ*m. (h) Hyphal structures extruding from stomata on the adaxial surface of a Bd21 leaf, 16 days following Rcc09B4 infection. Scale bars = 50 *μ*m. (i) Light microscope image of swan's neck-shaped *Ramularia collo*-*cygni* conidiophores, produced on the abaxial side of a Bd3-1 leaf blade exposed to high light treatment, stained with 0·01% aniline blue, 21 days following spray inoculation with Rcc09B4. Arrows indicate *R*. *collo*-*cygni* conidia. Scale bar = 20 *μ*m. (j–k) Comparison of the effect of low light intensity (j) compared to high light intensity (k) pretreatment on the growth of Bd21 (left-hand side) and Bd3-1 (right-hand side) plants. Scale bars = 1 cm.

**Figure 4:**
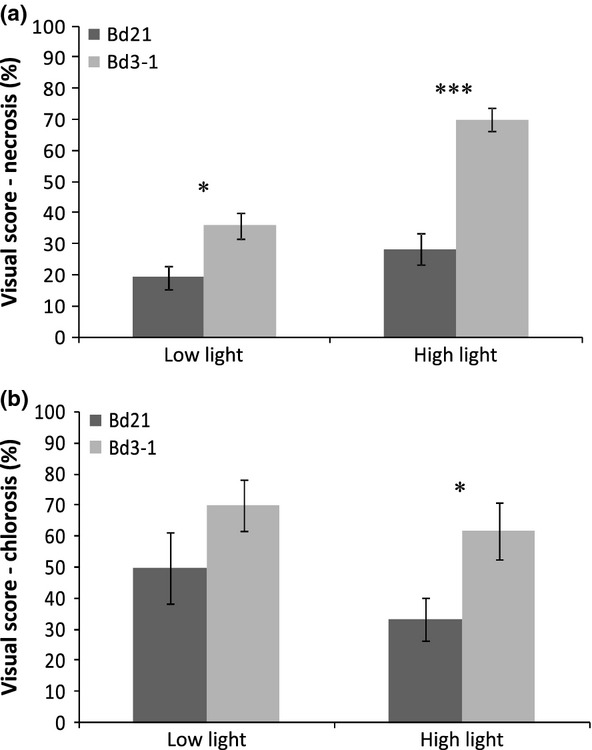
Ramularia leaf spot disease visual scoring system on *Brachypodium distachyon* Bd21 and Bd3-1 accessions grown under high light conditions and post-infection treatments with low or high light, 21 days following Rcc09B4 spray inoculation. (a) Visual disease scores of necrotic spots developed on Bd21 and Bd3-1 leaves incubated following spray inoculation in low or high light conditions. (b) Visual disease scores of chlorotic area developed on Bd21 and Bd3-1 leaves incubated following spray inoculation in low or high light conditions. Error bars indicate standard errors. ***P-value < 0·001; *P-value < 0·05.

Bd21 and Bd3-1 plants were grown under two light intensity regimes prior to inoculation with *R*. *collo*-*cygni* to test if RLS symptom expression is associated with light levels as occurs in its natural host barley (Makepeace *et al*., 2008). By 21 dpi with *R*. *collo*-*cygni*, leaves of both Bd accessions had developed brown necrotic spots surrounded by chlorotic areas (Fig.3c–f) which were reminiscent of RLS symptoms observed on barley under laboratory conditions (Fig.3a,b). Lesion size and frequency was greater on both accessions grown under the high light regime prior to inoculation (Fig.3c–f). The size of the necrotic lesions on Bd3-1 plants were greater with larger chlorotic areas surrounding the lesions than those observed on Bd21 plants grown under both light regimes (Fig.3c and 3e compared to 3d and 3f). Observation under a light microscope of the abaxial surface of aniline blue-stained Bd3-1 leaves revealed amber-brown colouration of adjacent cells surrounding the necrotic lesions. However, the lesions were not always obviously associated with the presence of *R. collo-cygni* hyphae (Fig.3g). Areas of dense hyphal colonization were observed on both sides of the leaf blade and, on the abaxial surface, were associated with hyphal structures emerging from stomata (Fig.3h). Also observed were the typical swan's neck-shaped conidiophores of *R*. *collo*-*cygni*. The conidiophores bearing conidia were observed emerging from the interstitial space between adjacent epidermal cells on the abaxial surface of the leaf blade (Fig.3i). Interestingly, the two light pretreatments resulted in plants with strikingly different plant morphologies irrespective of Bd accession. Bd plants grown for 3 weeks under the low light regime (150–200 *μ*mol m^−2^ s^−1^) had just begun to tiller at the time of inoculation and produced a low number of long, narrow, thin leaves (Fig.3j). In contrast, Bd plants grown under the high light regime (400–450 μmol m^−2^ s^−1^) produced several tillers and produced a greater number of shorter, broader leaves by the time of inoculation (Fig.3k). Visual disease scores were recorded 21 days following *R*. *collo*-*cygni* infection in a repeat experiment to assess the extent of foliar necrosis and chlorosis associated with RLS symptoms in Bd. Bd21 and Bd3-1 plants were grown under the high light regime before inoculation and either kept in high light or moved to the low light conditions after inoculation. Leaves of Bd3-1 plants had significantly more necrotic lesions than leaves of Bd21, whether on plants transferred to low light after inoculation (*P *=* *0·014) or on plants held at high light after inoculation (*P *<* *0·001). The post-inoculation light treatment did not result in a significant difference (*P *=* *0·17) in the amount of necrotic lesions observed on Bd21 leaves. However, Bd3-1 leaves developed significantly more necrotic lesions (*P *<* *0*·*001) when exposed to high light compared to low light treatment (Fig.4). The development of leaf chlorosis associated with RLS infection did not differ between the two Bd accessions when transferred to low light after inoculation (*P *=* *0·186) but leaf chlorosis was significantly higher on leaves of Bd3-1 plants than on leaves of Bd21 when transferred to the high light regime (*P *=* *0·033; Fig.4). The severity of chlorotic symptoms was not affected by the post-inoculation light regime for either accession (Fig.4).

## Discussion

*Brachypodium distachyon* has been proposed as a model system to enhance research into the biology of grasses and cereals (Draper *et al*., 2001; Brkljacic *et al*., 2011). One such area of research where Bd has shown potential as a useful model is in the area of plant–pathogen interactions. Studies have indicated that Bd can be used to examine the interactions and host responses to both fungal and viral pathogens of cereal crops such as wheat and barley (Draper *et al*., 2001; Routledge *et al*., 2004; Barbieri *et al*., 2011; Peraldi *et al*., 2011; Cui *et al*., 2012). The purpose of this study was to determine if Bd can be used as a model to investigate plant–pathogen interactions with the cereal pathogens *Oculimacula* spp. and *R*. *collo*-*cygni*, the causal agents of eyespot and RLS diseases, respectively. The results clearly demonstrate that these pathogens form a compatible interaction with Bd, thus extending the range of cereal pathosystems that can be investigated with this model.

Stem base infection of both Bd accessions with *Oculimacula* spp. resulted in the development of eye-shaped, honey-brown to dark brown lesions similar to those observed in wheat. Eyespot symptoms were also observed on internal leaf sheaths, indicating that the fungus can penetrate successive tissue layers, as occurs in wheat and barley. The multicellular aggregations of hyphal structures closely resembled the infection plaques formed by *O. yallundae* on wheat as described by Daniels *et al*. (1991) from which the fungus penetrates the underlying cells. The presence of ‘pin-holes’ beneath the infection plaque hyphal structures also closely resemble the penetration holes observed on wheat (Daniels *et al*., 1991). Examination by scanning electron microscopy would be required to establish whether *O. yallundae* also produces matrix collars about the penetration points as is the case in wheat (Daniels *et al*., 1991). In addition to direct penetration of host tissue it appeared that *O. yallundae* also attempted entry of Bd leaf sheaths through stomatal cavities by adventitious hyphae.

Inoculation of Bd with hyphal fragments of *R*. *collo*-*cygni* resulted in the development of foliar symptoms highly similar to those observed on the natural host, barley (Walters *et al*., 2008). The compatibility of the interaction between Bd and *R*. *collo*-*cygni* was confirmed by light microscopy of infected leaf tissues. The characteristic hyphal swellings emerging from stomatal cavities on the abaxial surface of the leaf epidermis combined with the production of conidia-bearing conidiophores that exhibit the swan's neck shape typical of *R*. *collo*-*cygni* (Sutton & Waller, 1988) demonstrates that this fungus is able to complete its life cycle on Bd. Both the necrotic and chlorotic symptoms on leaves of plants grown under high light conditions prior to inoculation covered a larger leaf area, regardless of the Bd accession, than on leaves of plants grown under low light conditions. These results concur with those of Makepeace *et al*. (2008) who reported that high light treatment of barley plants before inoculation followed by reduced light levels after inoculation produced the greatest level of disease symptoms, as was observed for the two Bd accessions examined in this study. Here, a difference was observed in symptom development between the two Bd accessions where plants were exposed to high light before inoculation and placed under either the same high light level or a reduced light level post-inoculation. The relationship between RLS and light levels appears complex. Necrotic symptoms were greater in Bd3-1 if maintained at high light following inoculation whereas no difference was observed in Bd21. Chlorotic symptoms were unaffected by post-inoculation light regime for either accession. Again, these findings mirror those reported for barley. Makepeace *et al*. (2008) reported that, for plants treated with high light prior to inoculation, symptoms on most of the nine varieties tested were less on leaves held at high light after inoculation than those transferred to low light. However, this was not the case for all the varieties tested. The varieties Chariot and Pallas exhibited greater RLS symptoms when held under high light than when transferred to low light after inoculation, while in line P22 symptoms were not influenced by the post-inoculation light regime (Makepeace *et al*., 2008). Thus the effect of different light regime intensities on RLS symptom development varies both among Bd accessions and for barley varieties. Further evaluation of the effect of light on this disease in additional Bd accessions would be required to appraise how useful Bd could be as a model to investigate the complex interaction between pathogen, genotype and environment. *Ramularia collo-cygni* is generally described as a specific pathogen of spring and winter barley. However, Walters *et al*. (2008) mentioned reports of *Ramularia* infection observed on oats and wheat. Therefore, observation of a compatible interaction between *R. collo-cygni* and Bd supports the view that the pathogen may have evolved a wider range of compatible hosts than previously suspected.

Interestingly, Bd3-1 was shown to be more susceptible than Bd21 to both *Oculimacula* spp. and *R*. *collo*-*cygni* infection. A similar difference between the two accessions was previously reported for susceptibility of foliar and floral tissues to *Fusarium graminearum* and in susceptibility to the fungal mycotoxin deoxynivalenol (Peraldi *et al*., 2011). Overall, results from these three different pathosystems suggest there may be a difference between Bd3-1 and Bd21 accessions in basal or broad-spectrum resistance to fungal pathogens. Cui *et al*. (2012) recently used a recombinant inbred population derived from a cross between Bd21 and Bd3-1 accessions to fine-map the *Bsr1* resistance gene to *Barley stripe mosaic virus* (BSMV). These authors reported that Bd3-1 was incompatible to BSMV infection while Bd21 was susceptible, a reverse of the situation for resistance to the three fungal hemibiotrophs studied to date (Peraldi *et al*., 2011; present study). In future studies it is intended to employ this population to undertake a quantitative genetic analysis of the response to the three fungal pathogens. This will establish whether the response to the three diseases is due to a common mechanism or resistance to the three is independently inherited.

In conclusion, this work provides the first report documenting a compatible interaction between Bd and the causal agents of eyespot and RLS diseases. This work therefore extends the range of pathogens compatible with Bd and further supports the view that this grass model plant is a powerful tool with which to conduct research on plant–pathogen interactions that are relevant to small-grain cereals. Previously, work using Bd provided the first demonstration of infection of intact foliar tissues by *Fusarium* species and identified a novel route for initial infection at the base of macrohairs (Peraldi *et al*., 2011). No such direct penetration of intact foliar tissues by *F. graminearum* had been observed previously but, following the study on Bd, foliar infection was observed in wheat (Wagacha *et al*., 2012), demonstrating the ability of the Bd pathosystem to provide genuine insight to advance the understanding of the pathogen's interaction with its crop host. Furthermore, two T-DNA insertional mutant collections are currently available (Vain *et al*., 2008; Bragg *et al*., 2012, using Bd21 and Bd21-3 accessions, respectively) and could be used to screen for altered resistance to RLS or eyespot diseases in Bd. The results presented herein further support the view that Bd can be used to conduct functional genetic research aimed towards model-to-crop translation of agronomically important disease resistance traits.
